# Inferring pesticide toxicity to honey bees from a field‐based feeding study using a colony model and Bayesian inference

**DOI:** 10.1002/eap.2442

**Published:** 2021-09-05

**Authors:** Jeffrey M. Minucci, Robert Curry, Gloria DeGrandi‐Hoffman, Cameron Douglass, Kris Garber, S. Thomas Purucker

**Affiliations:** ^1^ Office of Research and Development Center for Public Health and Environmental Assessment U.S. Environmental Protection Agency 109 TW Alexander Drive Durham North Carolina 27709 USA; ^2^ Crystal River Consulting LLC 1909 Stonecastle Drive Keller Texas 76262 USA; ^3^ USDA‐ARS Carl Hayden Bee Research Center 2000 East Allen Road Tucson Arizona 85719 USA; ^4^ USDA‐Office of Pest Management Policy 1400 Independence Avenue SW Washington D.C. 20250 USA; ^5^ Office of Pesticide Programs U.S. Environmental Protection Agency 1200 Pennsylvania Avenue NW Washington D.C. 20460 USA; ^6^ Office of Research and Development Center for Computational Toxicology and Exposure U.S. Environmental Protection Agency 109 TW Alexander Drive Durham North Carolina 27709 USA

**Keywords:** approximate Bayesian computation, Bayesian inference, colony feeding study, colony models, Honey bees *(Apis mellifera)*, pesticides, risk assessment

## Abstract

Honey bees are crucial pollinators for agricultural crops but are threatened by a multitude of stressors including exposure to pesticides. Linking our understanding of how pesticides affect individual bees to colony‐level responses is challenging because colonies show emergent properties based on complex internal processes and interactions among individual bees. Agent‐based models that simulate honey bee colony dynamics may be a tool for scaling between individual and colony effects of a pesticide. The U.S. Environmental Protection Agency (USEPA) and U.S. Department of Agriculture (USDA) are developing the VarroaPop + Pesticide model, which simulates the dynamics of honey bee colonies and how they respond to multiple stressors, including weather, *Varroa* mites, and pesticides. To evaluate this model, we used Approximate Bayesian Computation to fit field data from an empirical study where honey bee colonies were fed the insecticide clothianidin. This allowed us to reproduce colony feeding study data by simulating colony demography and mortality from ingestion of contaminated food. We found that VarroaPop + Pesticide was able to fit general trends in colony population size and structure and reproduce colony declines from increasing clothianidin exposure. The model underestimated adverse effects at low exposure (36 µg/kg), however, and overestimated recovery at the highest exposure level (140 µg/kg), for the adult and pupa endpoints, suggesting that mechanisms besides oral toxicity‐induced mortality may have played a role in colony declines. The VarroaPop + Pesticide model estimates an adult oral LD_50_ of 18.9 ng/bee (95% CI 10.1–32.6) based on the simulated feeding study data, which falls just above the 95% confidence intervals of values observed in laboratory toxicology studies on individual bees. Overall, our results demonstrate a novel method for analyzing colony‐level data on pesticide effects on bees and making inferences on pesticide toxicity to individual bees.

## Introduction

Honey bees (*Apis mellifera* L.) provide essential pollination services for many agricultural crops, but these services are threatened by increasing colony losses in North America and Europe in recent decades (Tylianakis 2013, Potts et al. 2016). While multiple stressors (disease, nutrition, genetics, and chemicals) are implicated, pesticides may be important contributors to these declines (National Honey Bee Health Stakeholder Conference Steering Committee 2012, Goulson et al. 2015) because they can cause direct mortality to individual bees, as well as a range of sublethal effects (Krupke et al. 2012, Siviter et al. 2018) and have frequently been found in colonies (Mullin et al. 2010, Traynor et al. 2016, Tosi et al. 2018). Linking the effects of pesticides on individual bees to whole‐colony success or failure is challenging because colonies are complex systems (i.e., superorganisms) with emergent properties derived from internal population dynamics and complex interactions among individuals (Seeley 1995, Camazine et al. 2003, Godfray et al. 2014). While it is possible to measure declines in colony‐level properties over time (e.g., number of adult bees and cells of honey), it is difficult to observe effects of pesticides inside hives at the individual bee level and directly link individual‐level and colony‐level effects. Agent‐based models that simulate internal colony population dynamics in response to pesticide exposure may allow inference on how pesticide effects on individual bees scale up to colony growth and survival.

Many managed honey bee colonies are located in or near agricultural areas, leading to exposure to pesticides being applied to control crop pests (Mullin et al. 2010, Tosi et al. 2018). Two primary routes of pesticide exposure for honey bees have been identified: contact and oral. Contact exposure with pesticides occurs when foraging bees are directly sprayed or when they land on foliage that has received direct spray or drift (Girolami et al. 2012, Krupke et al. 2012). Oral exposure occurs through ingestion of pollen or nectar derived from either pesticide‐treated agricultural crops (Girolami et al. 2009, Krupke et al. 2012) or from neighboring wild plants contaminated through drift or transfer through soil and subsequent root uptake (Krupke et al. 2012, USEPA et al. 2014, Bonmatin et al. 2015, Botías et al. 2016, Mogren and Lundgren 2016). Furthermore, some classes of pesticides are relatively stable in the environment, leading to soil contamination that can persist for months or years after application (Goulson 2014, Jones et al. 2014). Acute and chronic pesticide exposure to honey bees can lead directly to bee mortality, or indirectly via sublethal effects such as reduced foraging success and flight ability (Henry et al. 2012, Fischer et al. 2014, Tosi et al. 2017, Morfin et al. 2019*a*), impaired olfactory learning (Decourtye et al. 2005, Williamson and Wright 2013, Siviter et al. 2018, Morfin et al. 2020), and increased susceptibility to diseases (Alaux et al. 2010, Di Prisco et al. 2013, Doublet et al. 2015).

Over the past decade, regulatory agencies such as the U.S. Environmental Protection Agency (USEPA) and European Food Safety Authority (EFSA) have developed guidance for risk assessors and stakeholders on data needs for honey bee toxicity testing, as well as how to evaluate of potential risk of pesticides to bees (EFSA 2013, USEPA et al. 2014, Rortais et al. 2017). These efforts were partly a response to public concern over significant losses of honey bee colonies in the United States and Europe (National Research Council 2007, Oldroyd 2007), and also due to development of increasingly reliable laboratory toxicity testing protocols for honey bees.

USEPA’s process for assessing risk to bees utilizes a tiered approach that begins with acute and chronic testing of individual adults and individual larvae, and in higher tiers, considers exposures and effects to colonies. In Tier I toxicity studies, individual larval or adult bees are exposed to a single contact or oral dose (acute toxicity studies) or repeated oral doses (chronic toxicity studies) of a given pesticide. These studies derive standard toxicity endpoints based on apical endpoints (survival, growth, or reproduction) that can be compared to estimated environmental exposures. Acute exposure endpoints based on mortality are represented by median lethal doses (LD_50_ values), while chronic exposure toxicity endpoints are represented by Lowest Observed Adverse Effect Concentrations (LOAECs) and No Observed Adverse Effect Concentrations (NOAECs). These values are compared to Estimated Environmental Concentrations (EECs) for contact and oral exposures of a given pesticide that are generated using the BeeREX model (USEPA et al. 2014). These comparisons, represented by risk quotients (RQs), are then compared to the Levels of Concern (LOCs) for acute and chronic toxicity, 0.4 and 1.0, respectively, which were established by the USEPA to define whether there is a potential risk concern for effects to individual bees (USEPA 2012).

Based on the results of Tier I studies, Tier II testing may be conducted. Tier II studies involve comparison of empirically based concentrations of pesticides in pollen and nectar to results of controlled colony‐level toxicity studies (colonies are fed known concentrations of a pesticide), as well as consideration of effects to colonies exposed in semi‐field conditions (tunnel or enclosure studies). If there are risk concerns (adverse colony‐level effects at empirically observed concentrations of pesticides) from the more controlled Tier II studies, full‐field (Tier III), colony studies may be needed (USEPA et al. 2014). Semi‐ and full‐field studies evaluate pesticide toxicity at the colony‐level, including potential measurement of adverse effects on sublethal honey bee behavior such as foraging activity, and quantification of toxicity effects on honey bee brood and food production. Higher‐tier studies are considered more representative of real field exposures by honey bee colonies, but interpretation of their data can be confounded by interactions with other environmental influences and stressors (e.g., disease, nutrition, and parasites) and variability among colonies, and they are logistically challenging and expensive to conduct (USEPA et al. 2014). Models are therefore being developed in the United States and EU to simulate colony‐level effects of pesticides to aid synthesis of colony‐level data, and to provide regulatory agencies with additional evidence of whether higher tier (Tiers II or III) studies may be informative.

The U.S. model under development is VarroaPop + Pesticide (hereafter, VarroaPop), an age‐structured, agent‐based colony simulation model (Kuan et al. 2018). It was first developed to simulate colony growth and development through time (BEEPOP; DeGrandi‐Hoffman et al. 1989), and subsequently extended to include infestation by parasitic *Varroa* mites (*Varroa destructor*; DeGrandi‐Hoffman and Curry 2005) and pesticide exposure (Kuan et al. 2018) to determine their cumulative effects on colony growth and survival. Pesticide contamination of pollen and nectar can be calculated based on application method, similarly to the Tier 1 BeeREX model, or directly specified. Individual food consumption rates for each age and caste of bees are used to scale up exposure to the entire colony. Toxicity is applied to each day‐cohort, based on the logistic Hill equation with LD_50_ and slope parameters (Hill 1910).

Here, we present a method for inferring individual‐level pesticide toxicity from colony‐level field data, employing the VarroaPop + Pesticide agent‐based colony model. We used data from a registrant‐submitted feeding study on clothianidin, a nitroguanidine‐substituted neonicotinoid insecticide, in which colonies were dosed with spiked nectar of varying concentrations over a five‐week period. Because nectar contaminated with the active ingredient was provided directly to colonies, this study focuses only on dietary exposure routes. We did not evaluate the effect of *Varroa* mites because colonies were treated for *Varroa* and mite levels remained relatively low throughout the study. We implemented a Bayesian hierarchical model based on VarroaPop to explain dynamics of single colonies in the feeding study. We then applied Approximate Bayesian Computation (ABC) to fit our model to the empirical data and inferred parameters describing individual toxicity in VarroaPop. We hypothesized that (1) VarroaPop can explain general trends in colony population size observed in the control colonies and (2) individual‐level oral toxicity is sufficient to explain colony declines observed at high concentrations of clothianidin in the feeding study.

## Methods

### Colony feeding study design

A study on the effects of clothianidin on honey bee colonies in field conditions took place between 17 June 2014 and 27 April 2015 in the Piedmont region of North Carolina. Four *Apis mellifera ligustica* sister breeder queens were purchased from a commercial bee supplier (The Carolina Honey Bee Company, Travelers Rest, South Carolina, USA) and used to generate queens for the study colonies. Eighty‐four colonies were divided into 12 sites in low‐agriculture areas of Guilford, Randolph, Alamance, and Chatham counties. According to the USDA National Agricultural Statistics Service Cropland Data Layer, 8% of the land cover within 5‐mile radii of the apiaries was agricultural (Louque 2016; 1 mile = 1.6 km). Colonies were assigned to one of six treatment groups that received supplemental nectar feedings spiked with clothianidin at 0, 10, 20, 40, 80, and 160 µg/L. Measured clothianidin concentrations in the supplemental nectar were found to be slightly lower than the nominal treatment levels, so we refer to the treatments by the measured values, in units of µg/kg: 0, 10, 19, 36, 72, and 140 µg/kg. We also used these measured values as the pesticide inputs to VarroaPop. Clothianidin concentrations observed in crop nectar following foliar, soil, and seed applications range from 4 to 3,400 µg/kg, 4 to 40 µg/kg, and 1 to 4 µg/kg, respectively, and represent a variety of locations, conditions, and crops (USEPA 2017). However, the colony feeding study was designed to cover a wide range of exposure levels to determine the no observed adverse effect concentration (NOAEC) and lowest observed adverse effect concentration (LOAEC) and was not intended to replicate a specific crop application scenario. Treatments were assigned with a stratified random approach that standardized colony size (number of adult bees) among the treatments. Colonies were divided into 12 groups of 7, with the 7 largest colonies assigned to the one group, then the next 7 largest colonies, and so forth. Each group of colonies was then randomly assigned to either a treatment level (one per level per group) or the control (two per group). In total, there were 24 replicate colonies for the 0 µg/kg control and 12 replicates for each of the five treatment levels. Supplemental nectar feeding occurred continuously for 34 d from 26 June 2014 to 30 July 2014, with clothianidin content prescribed by treatment level. In addition to the supplied nectar, bees were allowed to forage naturally for pollen.

Colonies were checked for visible symptoms disease or pests, such as *Nosema*, foulbrood, *Varroa* mites and small hive beetles during each colony assessment, with no observations reported. The number of mites per 100 bees and *Nosema* spores per bee were quantified before nectar feedings began (18–23 June) and one week post‐feedings (5–11 August). No assays for other brood diseases or pests were performed. All colonies were treated for *Varroa* mites with an application of thymol in September in accordance with typical apicultural practice for the region (Louque 2016).

The condition of each colony was assessed before nectar feedings began (18–23 June), once during the feedings (15–18 July), and 1, 5, and 11 weeks post‐feeding (5–11 August, 8–12 September, and 14–22 October, respectively). All colonies survived until the October assessment period, however, two colonies from the 36 µg/kg treatment group were removed from the study due to technical errors. No colonies exhibited swarming behavior during the study period. During each colony condition assessment, hives were opened, and each frame was removed and inspected. Area coverage was measured for adult bees, larvae, pupae, eggs, honey, nectar, and pollen (bee bread). Area measurements were then converted to individual or cell counts, using density of adult bees (for adults) or density of cells on the frame (for all other endpoints) empirically measured in the study. To parameterize the initial nectar and pollen store parameters in VarroaPop + Pesticide, we converted nectar and pollen cell counts to weight, using cell depth of 12.5 mm, nectar density of 1.13 g/mL (30% sucrose solution), and pollen density of 1.45 g/mL (corn pollen, a major pollen source for the study colonies; Aylor 2002). Data used in our analysis (replicate‐level means and standard deviations) are publicly available (Louque 2016).

### VarroaPop + Pesticide model

The complete structure and equations of VarroaPop are described elsewhere (DeGrandi‐Hoffman et al. 1989, DeGrandi‐Hoffman and Curry 2004, 2005, Kuan et al. 2018); here, we provide a brief summary. VarroaPop is an agent‐based model that simulates colony dynamics, based on queen egg‐laying rate, development of workers and drones, and activity patterns of foragers. These dynamics are optionally modified by *Varroa* mite infestation (not considered here), and oral and/or contact exposure to pesticides. Queens are simulated as individual agents, with daily egg‐laying rate and proportion of eggs fertilized determined by weather, colony size, worker population, photoperiod, and fecundity (queen strength) (DeGrandi‐Hoffman et al. 1989). All other bees are simulated as day‐cohort agents that age and transition between life stages and consume pollen and/or nectar based on age and caste (Rortais et al. 2005, USEPA et al. 2014).

We focused our modeling on a time window spanning just before nectar feeding treatments began until 11 weeks post‐treatment. VarroaPop + Pesticide requires daily weather data on temperature, precipitation, hours of daylight, and wind speed for the simulation period to determine potential foraging time. We used National Oceanic and Atmospheric Administration (NOAA) weather data gridded at 0.25° × 0.25° resolution and centered at 35.875° N, 79.375° W near the center of the feeding study area (Fry et al. 2016). Mean daily temperature during the exposure period was 23.3°C, close to the 15‐yr average for this period of 23.9°C. Mean daily precipitation was 0.24 cm/d, 33% lower than the 15‐yr mean of 0.36 cm/d. In the model, when weather is favorable (maximum temperature between 12°C and 43.3°C, wind speed <21.1 m/s, daily rainfall <0.5 cm), foraging honey bees collect pollen and nectar from an infinitely large range area, based on a specified number of trips per day. Resources collected in excess of daily consumption are stored and potentially consumed later when daily foraging does not meet colony food requirements. Pesticide contamination of pollen and nectar can be calculated based on application method and timing, or directly specified (as in this study; Kuan et al. 2018). Oral dose is calculated by multiplying age‐ and caste‐specific daily pollen and nectar consumption rates by the concentration of pesticides present in each food resource. Mortality due to ingestion of this contaminated pollen/nectar is calculated for larval and adult age‐cohorts, based on the dose consumed and a logistic dose‐response curve (parameterized by LD_50_ and slope; Hill 1910, Kuan et al. 2018). Contact exposure to foraging bees can also be simulated in pesticide foliar spray scenarios, but is not considered in this study, which included only dietary exposure.

### Modeling the feeding study data using VarroaPop + Pesticide

We defined a Bayesian hierarchical model, which included the VarroaPop + Pesticide agent‐based model, to explain dynamics of single colonies in the feeding study. We then used it to simulate the 84 colonies in the feeding study and produce treatment by time point summary statistics that corresponded to observations in the study. We modeled the population structure of an individual colony *i* at a given time point *t* ($y_{i,t}$) as

(1) $$y_{i,t}\;=\;f\left(y_{i,\text{0}},x,z,\;{\mathrm{Init}}_{i},E_{i},{\mathrm{Str}}_{i},L_{i},t\right)$$

$${\mathrm{Str}}_{i\;}∼\mathrm{Normal}\left(\mu_{\mathrm{Str}},\sigma_{\mathrm{Str}}\right)$$

$$L_{i\;}∼\mathrm{Normal}\left(\mu_{L},\sigma_{L}\right)$$

$$\;x_{j\;}∼\mathrm{Unif}(a_{j},b_{j})\;\mathrm{for}\;j=1,2,\ldots ,n$$

$$\mu_{\mathrm{Str},\;L}∼\mathrm{Unif}\left(a_{\mathrm{Str},L}^{\mu },b_{\mathrm{Str},L}^{\mu }\right)$$

$$\sigma_{\mathrm{Str},\;L}∼\mathrm{Unif}\left(a_{\mathrm{Str},L}^{\sigma },b_{\mathrm{Str},L}^{\sigma }\right)$$

where *f* is the VarroaPop + Pesticide agent‐based model; **y**_*i*,0_ is initial population structure for colony *i;*, **x** is a vector of toxicity random variables; **z** is a vector of fixed variables including weather conditions; **Init** *_i_* is a vector of initial size, population structure, and food resources for colony *i*; *E_i_* is the clothianidin exposure level for colony *i*; and Str*_i_* and *L_i_* are random variables for queen strength (egg‐laying rate) and forager life span, respectively, for colony *i*. We considered Str*_i_* and *L_i_* to be random variables drawn from a normal distribution shared among all colonies in the study because they strongly influence population dynamics and vary between colonies (Kuan et al. 2018). Thus, our model uses these two random variables to account for the variance among replicate colonies in the feeding study. For the mean (μ) and standard deviation (σ) hyperparameters of the normal distributions, we defined uniform hyperpriors with μ bounded within [1, 5) for queen strength (equivalent to 1,000–3,000 eggs/d) and [4, 16) d for forager life span, the full range of possible values in VarroaPop, and σ within [0, 2) and [0, 3), respectively (Table 1). We also defined prior probability of toxicity parameters **x** as a uniform distribution spanning the range of plausible values (Table 1). Adult and larva oral LD_50_ was defined within [0.1, 100) ng/bee, a considerably wider range than that observed in laboratory studies (USEPA 2017). Adult and larva dose‐response curve slope was defined within [1, 9) percent mortality per ng clothianidin, the range defined in a previous sensitivity analysis of VarroaPop (Kuan et al. 2018). We then used Bayesian inference to estimate the joint posterior probability of toxicity parameters **x** and hyperparameters μ_Str_, σ_Str_, μ_L_, and σ_L_.

**Table 1:** List of parameters considered to be random variables and inferred through Approximate Bayesian Computation.

Parameter name in VarroaPop	Description	Units	Type	Lower limit	Upper limit
ICAdultLD50	oral LD_50_ for adults	ng/bee	prior	0.1	100
ICAdultSlope	slope of the adult dose‐response curve	mortality %/ng	prior	1	9
ICLarvaLD50	oral LD_50_ for larvae	ng/bee	prior	0.1	100
ICLarvaSlope	slope of the larva dose–response curve	mortality %/ng	prior	1	9
ICForagerLifespan (mean)	mean life span of foragers	d	hyperprior	4	16
ICForagerLifespan (SD)	SD of forager life span	d	hyperprior	0	3
ICQueenStrength (mean)	mean queen strength (∝ egg laying rate)	unitless	hyperprior	1	5
ICQueenStrength (SD)	SD of queen strength (∝ egg laying rate)	unitless	hyperprior	0	2

In addition to initial colony conditions, weather data, and toxicity parameters, VarroaPop + Pesticide requires parameterization of pollen and nectar foraging behavior and consumption rates for each life stage. We treated these as known constants shared among all colonies in the feeding study (Appendix S1: Table S1). For pollen and nectar consumption rates, we used the empirically derived values compiled in the USEPA Final Guidance on Bee Risk Assessments document, taking the mean when ranges were given (USEPA 2014). For the number of nectar‐gathering trips per day, we started with 10/d, the mean value used by the USEPA Bee Risk Assessment Framework document (USEPA 2012) and increased this until nectar stores could be maintained in VarroaPop for control treatment colonies. This resulted in a final value of 17 trips/d, which is within the previously reported range for foraging honey bees (Winston 1987). Because pollen foraging occurs primarily during the first half of the day, we set the number of pollen trips to 8 trips/d, which is close to the previously reported mean pollen foraging activity by honey bees (Klein et al. 2019).

To confirm that our model could fit the general population structure of the control colonies, we did an initial VarroaPop + Pesticide run with the mean initial conditions of the control and previously described parameters. We observed that, although the adult population count estimated by the model was in agreement with empirical data from the study, there were more pupae, larvae and eggs in the empirical data than was predicted by VarroaPop, suggesting unexplained mortality as pupae transition to adults. We therefore reduced the pupa‐to‐adult transition survival rate in the model from 100% to 60% (Appendix S1: Table S1), the level at which the predicted population structure roughly matched the control data.

### Model inference using Approximate Bayesian Computation with sequential Monte Carlo

We used Approximate Bayesian Computation (ABC) to infer probability distributions of toxicity parameters in our model, given the empirical feeding study data. ABC is a computational method for approximating the joint posterior probability distribution of a model by comparing its outcome to empirical data (either with individual data points or summary statistics) (Beaumont 2010, Csilléry et al. 2010). We compared the mean and standard deviation of empirical and estimated colony endpoints (number of adults, pupae, larvae, and eggs) for each treatment group by time combination. Parameter sets (particles) are either accepted or rejected based on whether their distance from the real data, as calculated by a distance function on summary statistics (in this case the sum of absolute deviation), is less than an acceptance criterion ɛ. A key advantage of ABC is that this distance function replaces a formal likelihood function, allowing inference on black‐box or agent‐based models like VarroaPop, which lack a tractable likelihood function.

To explore parameter space and propose potential parameter sets for ABC, we used a sequential Monte Carlo (SMC) algorithm, also known as particle filtering (Sisson et al. 2007, Toni et al. 2009, Doucet and Johansen 2011). This algorithm uses Monte Carlo iterations (called populations), each of which takes the distribution of particles accepted by ABC in the last population as the prior distribution from which to sample. With each successive population, the acceptance criterion ɛ is decreased, resulting in an increasingly close approximation of the posterior.

To carry out ABC with SMC sampling (ABC‐SMC), we used the pyABC package version 0.9.2 in Python 3.6 (Klinger et al. 2018), with computation distributed across 96 cores. We used the sum of absolute deviation (*L*_1_ norm) as the distance function because it may be more robust to outliers than the commonly used sum of squared deviations (*L*_2_ norm), but performs similarly, overall (Prangle 2017). We chose to fit our model to the mean and standard deviation of adult and egg counts because these two endpoints should provide sufficient information to estimate all intermediate life stages. Thus, our distance function compared model predictions and empirical data for 96 summary statistics (6 treatments × 4 dates × 2 endpoints × 2 statistics). For the transition function, which converts each set of accepted particles to the prior for the next population, we chose a local multivariate Gaussian kernel density estimator (KDE), using the nearest quarter of neighbors, which leads to faster convergence than a global KDE (Filippi et al. 2013). We adjusted ɛ each population to the median distance of accepted particles in the prior population. We ended sampling after 12 populations of 500 accepted particles because computational time had become prohibitive for such small return, since ɛ decreased only slightly with each additional generation and marginal posterior distributions had become stable.

### Predicting colony response to clothianidin

We used our fitted model to make predictions by sampling the joint posterior, which involved drawing parameters from our final generation of accepted particles, weighted by their distance from the empirical data, and evaluating the model using each parameter set to produce synthetic feeding study data. After 200 samples from the posterior, resulting in 200 model evaluations, we calculated the median value for the prediction of interest, as well as the percentiles corresponding to the 68%, 95%, and 99% prediction intervals (roughly 1, 2, and 3 standard deviations). These intervals reflect variation in individual colony strengths, as well as clothianidin toxicity parameters, as inferred from the empirical data. Because we sampled from the joint posterior, all predictions reflect the covariance structure of the parameters.

We used this method to predict the clothianidin adult and larva oral dose‐response curves, and distribution of egg‐laying rates (derived from queen strength) and forager life spans among colonies in the feeding study. We also predicted colony population structures through time for each treatment and compared these results to predictions for the control treatment. We then assessed whether our model predicted a significant reduction in the number of adults, pupae, larvae, and eggs at any time during the study, for each treatment level, as well as several untested exposure levels between 50 and 95 µg/kg. We defined a significant reduction as a period when the predicted difference from the control was below zero, within three different levels of confidence using the 68%, 95%, and 99% prediction intervals.

## Results

### Details of ABC‐SMC sampling

**Fig. 1:**
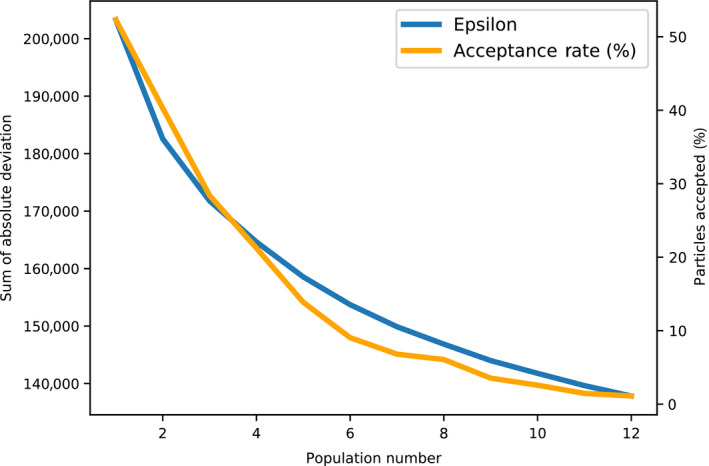
The acceptance threshold ɛ (blue) and acceptance rate (yellow) of Approximate Bayesian Computation with Sequential Monte Carlo sampling through 12 populations.

**Fig. 2:**
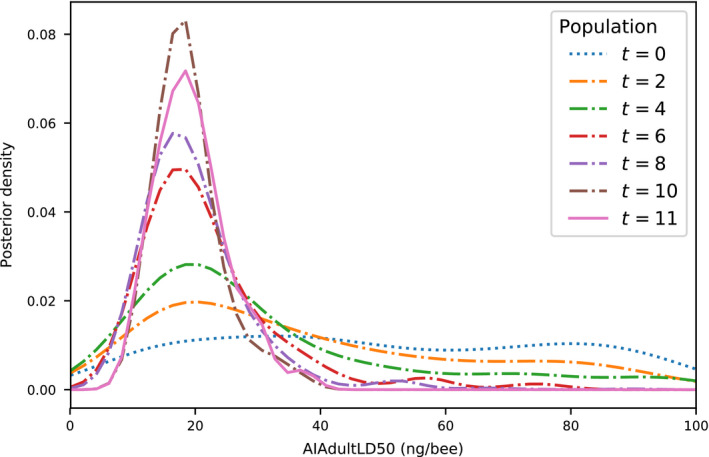
The posterior probability density of the adult oral LD_50_ parameter through 12 populations (*t*) of Approximate Bayesian Computation with Sequential Monte Carlo sampling.

We used Approximate Bayesian Computation with sequential Monte Carlo sampling (ABC‐SMC) to infer posterior probability distributions of key parameters in our VarroaPop + Pesticide‐based statistical model. Sampling occurred over 12 populations, with acceptance rates that began at 52.3% and decreased to 1.1% (Fig. 1). The total number of parameter sets (particles) considered was 144,158, each of which required 82 individual runs of VarroaPop + Pesticide, for a total ˜11.8 million model runs. Actual computation time was ˜13 d using 96 cores. Sampling was stopped after population (SMC iteration) 12 due to increasingly long computation times yielding little improvement in the acceptance threshold ɛ (Fig. 1). At this point in the algorithm, there were no major shifts in the posterior probability of parameters between generations (Fig. 2).

### Comparison of model predictions to the empirical feeding study data

**Fig. 3:**
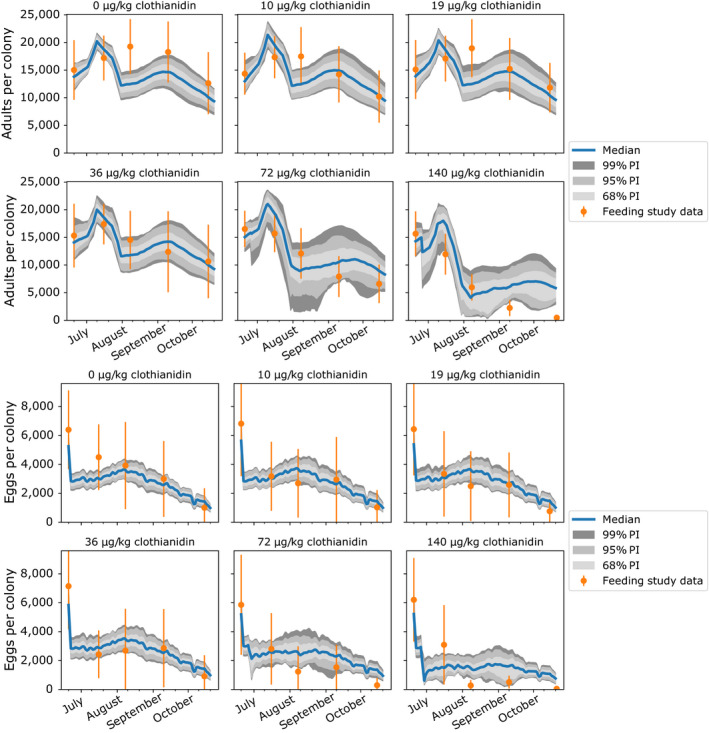
Predicted number of adults (top) and eggs (bottom) during the feeding study vs. empirical data (orange dots with lines showing standard deviation). Solid blue lines represent the median prediction and shaded regions denote the 68%, 95%, and 99% prediction intervals (PI). For pupae and larvae endpoints, see Appendix S2: Fig. S2.

To assess whether our VarroaPop + Pesticide‐based model could explain patterns of the feeding study, we compared our ABC‐SMC‐parameterized model’s predicted colony demographics through time to empirical data. For control treatment colonies, model predictions matched general temporal trends, which included a relatively stable adult population, sudden declines in pupae and adults at the final sampling point, and a consistent decrease in the number of eggs (Fig. 3; also see Appendix S2: Fig. S2). The model also successfully predicted declines across all population endpoints (counts of each caste) for the 72 and 140 µg/kg treatments, although it underpredicted the magnitude of decline at 140 µg/kg for adults, pupae and larvae. The 95% prediction intervals, which captured variability in parameter values and individual colony strength, overlapped with the standard deviation of the field data for adult bees in 21/24 treatments (87.5%) by sampling date combinations, excluding the initial time points.

### Predicted no/lowest observed adverse effect concentration (NOAEC/LOAEC)

**Fig. 4:**
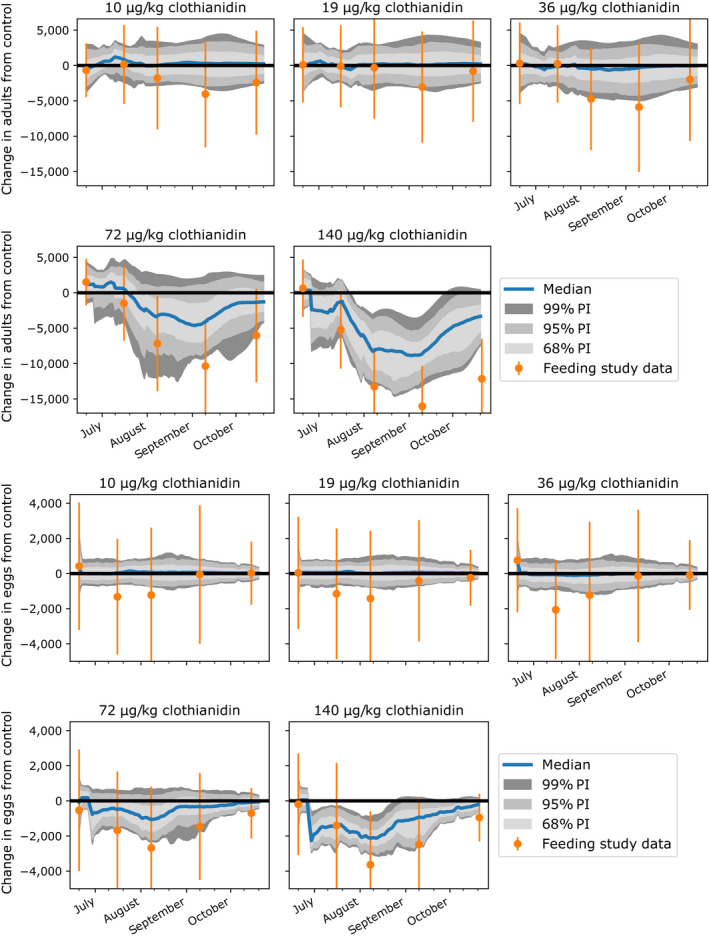
Predicted change in number of adults (top) and eggs (bottom) from the control. Empirical feeding study data is represented by orange dots. Solid blue lines represent the median prediction and shaded regions denote the 68%, 95%, and 99% prediction intervals. For pupae and larvae endpoints, see Appendix S2: Fig. S3.

**Fig. 5:**
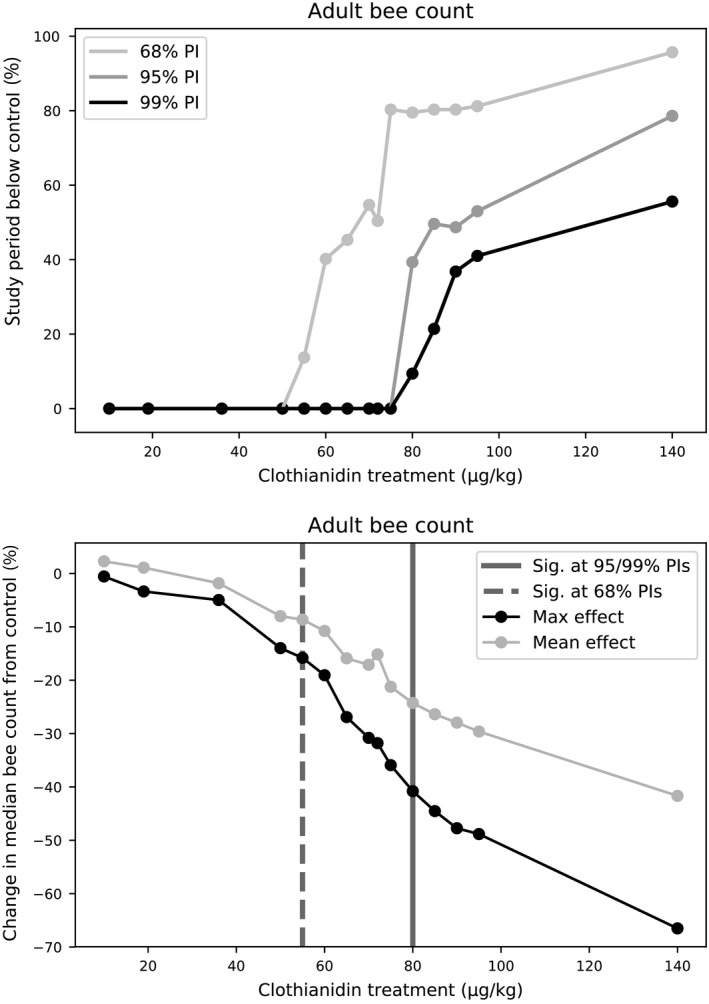
Percentage of the study period that each treatment was predicted to have a significant reduction in adult bee counts, compared to the control, for three different levels of confidence: 68%, 95%, and 99% prediction intervals (top), and the maximum and mean reduction in median adult bee counts for each treatment (bottom). The study period is the first day of treatment until the final colony condition assessment of 2014. Adult bee count was considered significantly reduced (compared to the control) when the prediction interval of the change did not contain zero. Clothianidin levels 50–70 µg/kg and 75–95 µg/kg were predicted by the model but were not present in the empirical feeding study.

We used our predicted colony size and population structure trajectories across the clothianidin treatments to estimate the no observed adverse effect concentration (NOAEC) and lowest observed adverse effect concentration (LOAEC) for adult, larvae, pupae and egg endpoints. When comparing all treatment levels present in the colony feeding study to the control, our model predicted a NOAEC and LOAEC of 36 and 72 µg/kg, respectively for the 68% prediction interval, and 72 and 140 µg/kg, respectively, for the 95% and 99% prediction intervals, on the basis of adverse effects on adults and brood (Fig. 4, Appendix S2: Fig. S3). According to the 95% prediction interval, colonies in the 140 µg/kg treatment had significant adult bee count reductions vs. the control for 78.6% of the study period (i.e., 78.6% of the time the 95% prediction intervals for change in number bees from the control did not contain zero; Fig. 5, top; Appendix S1: Table S2). The mean reduction in the median adult bee count for treatment colonies vs. control colonies over the study period was 1.8%, 15.2%, and 41.7% for the 36, 72, and 140 µg/kg treatments, respectively. The maximum reduction in the median adult bee count for treatment colonies vs. control colonies was 5.0%, 31.8%, and 66.5% for the 36, 72, and 140 µg/kg treatments, respectively (Fig. 5, bottom).

**Fig. 6:**
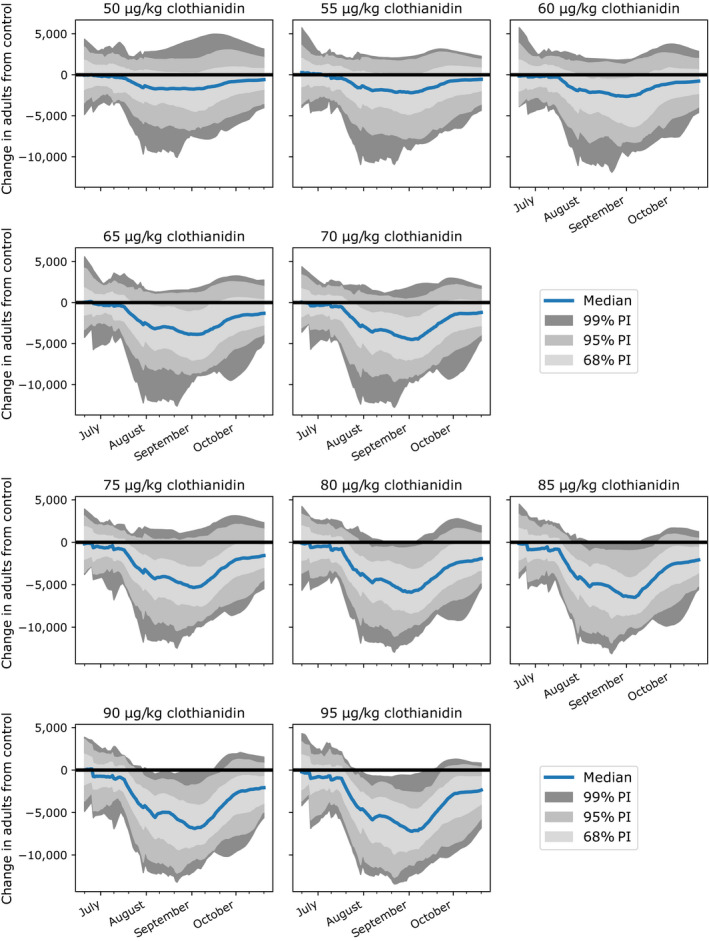
Predicted change, from the control, in number of adults for clothianidin levels not tested in the feeding study. Solid blue lines represent the median prediction and shaded regions denote the 68%, 95%, and 99% prediction intervals. For pupae, larvae, and eggs endpoints, see Appendix S2: Figs. S4, S5, S6.

Our parameterized model also allowed us to estimate a more precise significant effects threshold occurred than was possible in the colony feeding study, by predicting adverse effects at intermediate treatment levels that were not included in the feeding study design. We predicted effects on colonies from exposure to a range of 50–95 µg/kg clothianidin‐spiked nectar, using 5 µg/kg intervals. We found that a significant negative effect occurred at 55 µg/kg for the 68% prediction interval, and at 80 µg/kg for the 95% and 99% prediction interval, based on adverse effects on the number of adult bees (Fig. 6), and brood (Appendix S2: Figs. S4, S5, S6). According to the 95% prediction interval, colonies exposed to 75 µg/kg clothianidin did not have significant adult bee count reductions vs. the control, while those exposed to 80 µg/kg clothianidin had significant adverse effects for 39.3% of the study period, on average (Fig. 5, top; Appendix S1: Table S2). The mean reduction in the median adult bee count for treatment colonies vs. control colonies over the study period was 8.6% and 24.3% for the 55 and 80 µg/kg treatments, respectively. The maximum reduction in the median adult bee count for treatment colonies vs. control colonies was 15.8% and 40.8% for the 55 and 80 µg/kg treatments, respectively (Fig. 5, bottom).

### Probability distributions of model parameters inferred from feeding study data

Through ABC‐SMC, we inferred the most probable parameters for our model from empirical feeding study data. We considered four VarroaPop + Pesticide parameters that characterize pesticide toxicity at the individual bee level. Adult LD_50_, the median lethal oral dose for adults and foragers, had a large impact on our model’s ability to fit the empirical data and, therefore, had a sharply defined marginal posterior probability distribution (Appendix S3: Fig. S1). The median adult LD_50_ was 18.9 ng/bee, with a 95% credible interval (CI) of 10.1–32.6 ng/bee (Fig. 2). The slope of the adult oral dose‐response curve had a median value of 6.1 (95% CI, 2.2–8.8). In contrast with adult oral toxicity, larval toxicity did not show a strong marginal trend. The median larval LD_50_ was 29.3 ng/bee, with a 95% CI that covered most of the possible range (3.5–93.7 ng/bee), and median slope of the larva dose‐response curve was 5.5 (95% CI, 1.4–8.7).

We also inferred population‐level parameters that described the distribution of colony strengths across colonies in the feeding study (Appendix S3: Fig. S1). Queen strength, which controls the maximum egg‐laying rate in VarroaPop and varies from 1 to 5, had a mean of 3.3 (95% CI, 1.8–4.7) and a standard deviation of 1.8 (95% CI, 1.3–2.0). Forager life span, which varies from 4 to 16 d in VarroaPop, had a mean of 13.5 d (95% CI, 9.5–15.8 d) and a standard deviation of 1.7 d (95% CI, 0.2–2.9 d).

### Model predictions of clothianidin toxicity and colony strength

**Fig. 7:**
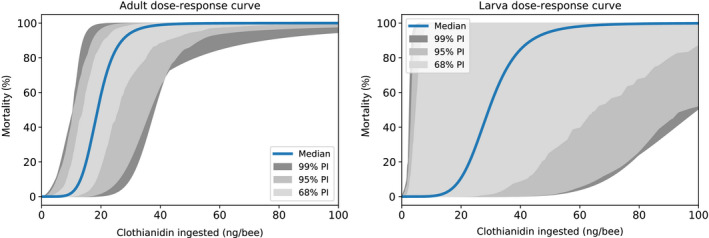
Our model’s predicted adult (left) and larva (right) dose–response curves, given the empirical feeding study data. Solid blue lines represent the median prediction and shaded regions denote the 68%, 95%, and 99% prediction intervals.

We used our parameterized model to infer clothianidin dose‐response curves that best explain the empirical feeding study data. The median adult oral dose‐response curve indicated that individual mortality, at a rate of at least 1%, began at 8.9 ng/bee and increased to 99% by 34.8 ng/bee (Fig. 7, left). Accounting for uncertainty in the adult LD_50_ and slope parameters, 95% of dose‐response curves exhibited at least 1% mortality at a dose of <18.3 ng/bee and reached at least 80% mortality by 45.5 ng/bee. The larva oral dose‐response curve was more variable due to greater uncertainty in the larva LD_50_ and slope parameters (Fig. 7, right). The median larva curve exhibited a mortality rate of at least 1% at 12.8 ng/bee and reached 99% at 67.3 ng/bee. Considering the variability in larva dose‐response curves, 95% of curves showed at least 1% mortality at a dose of <48.4 ng/bee and at least 52.6% mortality at 100 ng/bee.

We also inferred distributions of queen egg‐laying rates and forager life span among individual colonies, by sampling from the posteriors of the population‐level means and standard deviations. Queen egg‐laying rates varied widely from 1,000 to 3,000 eggs/d, but most fell between 1,750 and 2,500 eggs/d (Appendix S1: Fig. S1: left). In contrast, the distribution of forager life span among colonies was concentrated at the high end of the range, between 12 and 15 d (Appendix S2: Fig. S1: right).

## Discussion

Our study demonstrates that the VarroaPop colony simulation model can be successfully fit to empirical field data from colony‐level toxicity studies, providing novel inference on in‐hive dynamics. Because field‐based colony‐level studies are logistically and financially expensive, models like VarroaPop are a promising method for gaining additional information on colony‐level effects using input parameters from laboratory toxicity testing. Furthermore, colony simulation models can help separate effects of pesticides from factors like weather, temporal shifts in demography (e.g., population growth/reduction and change in structure), and colony‐to‐colony variation in queen egg‐laying rate. Our analysis of the clothianidin feeding study data suggests that acute oral toxicity to adult workers and foragers is sufficient to explain the majority of colony declines observed at 72 and 140 µg/kg, although additional mechanisms appeared to prevent population recovery at 140 µg/kg.

### Simulating honey bee colony population trends and structure with VarroaPop

The VarroaPop model, when parameterized to the feeding study data using ABC, was able to predict overall trends in colony population through time as well as general caste structure, supporting our hypothesis that the model could reproduce general trends in the data. Trends in control data that were predicted included an initial increase in the number of adult bees; a decline in the number of adults, pupae and larvae at the last time point; and a consistently decreasing number of eggs. Although VarroaPop fit overall data trends, there was a consistent deviation from empirical results. VarroaPop predicted an initial spike and recovery in the predicted adult and pupa populations, causing them to peak several weeks earlier than in the feeding study. This lagged response error is likely caused by initialization behavior of the VarroaPop model, which distributes all bees within each caste evenly, across all ages. In the feeding study, colony size was increasing at the beginning of the study period and it is likely that most bees were at the young end of their age ranges, leading to a later demographic peak as a function of pupae and adult development in the empirical data relative to the model (Page and Peng 2001). This behavior may be alleviated by allowing uneven distributions of bees across age ranges, or by obtaining data for a sufficiently long pre‐treatment period that allows the model to equilibrate to a natural age distribution based on egg‐laying‐rate.

The VarroaPop model also fit the general caste structure of the colonies in the feeding study. Both the empirical data and model predictions had a ratio of non‐forager adults : pupae : larvae around 2:2:1 for control colonies at all time points, except the final one. Interestingly, this ratio of non‐forager adults is 35–75% lower than the range predicted by a steady‐state model bee population using mortality rates from the literature (Torres et al. 2015). To fit this low number of adult bees, we reduced the VarroaPop pupa‐to‐adult transition survival rate to 60% from the default 100%. Lower adult bee population, relative to pupae and larvae, was likely caused by background mortality from sources other than clothianidin exposure.

One potential source of background mortality is infection by a honey bee pathogen (e.g., chalkbrood, foulbrood, or sacbrood virus) that may kill bees at the pupal stage (Aronstein and Murray 2010, Evans and Schwarz 2011), resulting in capped cells that may be counted in a census but fail to produce adults. While the authors of the colony feeding study did not observe these diseases (Louque 2016), they cannot be ruled out because colonies were not treated for any pathogens except *Varroa* mites. The authors did observe and quantify *Nosema* infection across all treatment colonies, however, and this pathogen causes reduced adult life span (Martín‐Hernández et al. 2011). Additionally, despite treatment for *Varroa* mites, *Varroa* presence was observed in study colonies, albeit at levels below the typical treatment threshold of >3 mites per 100 bees (0.71–2.40 mites per 100 bees in August 2014; Genersch et al. 2010, Honey Bee Health Coalition 2018). Mortality due to pathogen infection, in combination with *Varroa* pressure, may have contributed to poor overwintering success following the exposure period, which was noted in all treatments, including the control (Higes et al. 2008, Barron 2015).

### Using VarroaPop to explain the effect of clothianidin on colony endpoints

The VarroaPop model predicted declines in each colony‐level endpoint for the 72 and 140 µg/kg clothianidin treatments, with magnitudes similar to those in the feeding study. The model also predicted a subsequent recovery in the number of adults and pupae for these treatments to levels similar to the control colonies by the final colony condition assessment (11 weeks after exposure ended). This period of recovery in colony strength was not observed in the empirical data, where these colonies continued to decline through the end of the study period. Colonies in the feeding study also exhibited significant (relative to the control), but transient, adverse effects at 36 µg/kg for two of the four endpoints considered (adults and pupae), but our model did not predict this. Taken together, our results do not support our hypothesis that ingestion‐based toxicity is sufficient to explain colony declines in the clothianidin feeding study. Our model, which considered only acute oral toxicity, explained most of the negative effects seen in the empirical data and estimated a significant effect threshold of 80 µg/kg for all endpoints at 95% confidence, close to the empirical LOAEC of 72 µg/kg for eggs and larvae and within a factor of 3 of the empirical LOAEC of 36 µg/kg for adults and pupae. However, additional mechanisms may have contributed to declines at lower exposure levels (36 µg/kg) and lack of recovery at higher exposure levels (140 µg/kg).

Our model may have underestimated effects of 36 µg/kg clothianidin spiked nectar because it did not consider chronic, sublethal effects. Based on our estimated nectar consumption values (USEPA et al. 2014), 36 µg/kg translates to a daily exposure of 11.4%–26.7% of the inferred median LD_50_ (18.9 ng/bee) for adult workers, with some variation due to age. There is a growing understanding that prolonged exposure to neonicotinoid insecticides at concentrations below lethal doses can cause adverse effects in individual bees that could ultimately affect colony performance (Godfray et al. 2015). Sublethal exposure appears to inhibit immune response (Brandt et al. 2016) and may lead to greater susceptibility to pathogens (Di Prisco et al. 2013, Doublet et al. 2015) including *Nosema* (Alaux et al. 2010), a unicellular parasite observed across all treatments in the feeding study. Sublethal doses can also increase susceptibility to pathogens by inhibiting grooming and hygienic behaviors (Wu‐Smart and Spivak 2016, Morfin et al. 2019a, 2019b,*2019a*, *2019b*). In addition, sublethal doses may reduce foraging success (Yang et al. 2008) by inhibiting learning and memory (Decourtye et al. 2004, Williamson and Wright 2013, Tison et al. 2019, Morfin et al. 2020), navigation (Fischer et al. 2014, Stanley et al. 2015), and locomotor function (Williamson et al. 2014, Tosi et al. 2018). Future modeling efforts could include these sublethal effects pathways, in combination with *Varroa* mite pressure, to test whether addition of these mechanisms allows better prediction of transient colony declines at lower pesticide exposure levels. The model inference techniques applied here could be applied in combination with field data spanning a range of low exposure levels to guide development of these sublethal effects pathways within VarroaPop.

Sublethal effects may also explain the lack of recovery of the 72 and 140 µg/kg‐exposed colonies observed in the feeding study, but not predicted by our model. Although these effects may disappear by 11 weeks after exposure, increased colony disease burden (from decreased immunity) and decreased food stores (from altered foraging behavior) could lead to colony failures in the fall or winter (Higes et al. 2008, Naug 2009, vanEngelsdorp et al. 2009). Interestingly, colonies in the feeding study had a low number of workers, relative to pupae, across all treatments and time points. This low worker population, combined with clothianidin ingestion‐induced mortality and possible sublethal effects at 72 and 140 µg/kg, may have pushed colonies into failure. The VarroaPop model could better fit these scenarios by simulating pathogens in addition to *Varroa* mites; by including pesticide effects on immunity; and by adding feedback pathways critical to colony success such as thermoregulation, brood‐rearing, and hive defense capacity (Winston 1987, Stabentheiner et al. 2010, Barron 2015).

### Using a colony dynamics model to assess pesticide risk to bees

Fitting a honey bee colony dynamics model to field‐based experimental data allowed us to gain additional insights that could be leveraged as part of the risk assessment process used by regulatory agencies. Two key findings relative to the empirical colony feeding study were that some colony endpoints decline at lower exposure levels than our model predicted, and actual recovery of colony endpoints at the highest exposure level was less than our model predicted. As discussed above, these findings point to effects beyond oral toxicity‐induced mortality, suggesting that pathogen pressures and environmental variability also play important roles in colony‐level honey bee population dynamics.

We were also able to infer dose‐response relationships from the empirical data, endpoints that are typically difficult to estimate in whole‐colony studies without a predictive model that simulates internal colony processes. Our parameterized Bayesian model indicated that the median oral LD_50_ for adult bees in the feeding study was 18.9 ng/bee, which falls just above the range observed in laboratory acute oral toxicity studies on individual bees, 2.6–15.7 ng/bee (Laurino et al. 2011, USEPA 2017), and the 95% confidence interval of one of the two registrant‐submitted studies, 13.5–18.1 ng/bee (USEPA 2017). Interestingly, this suggests that Tier 1 individual bee toxicity experiments with clothianidin could be informative for adult oral toxicity in ecologically relevant scenarios, despite their inherent simplicity. In contrast, our analysis of the feeding study data provided little insight into the larval oral LD_50_, as evidenced by the broad probability distribution for this parameter, which did not improve as ABC‐SMC progressed, and there are no other studies that directly assessed acute larval toxicity (USEPA 2017). This highlights one drawback to highly parameterized inference methods: a data set can lack sufficient information to describe all parameters in a model (Luo et al. 2009). This issue of non‐identifiability can occur when parameters have functional interrelationships (correlation) (Li and Vu 2013), as in the case of larval and adult toxicity, where larval mortality leads to fewer adults and adult mortality leads to fewer larvae through reduced queen egg‐laying rate. In fact, the feedback of adult mortality on number of larvae may be responsible for the consistent and significant adverse effects predicted for larvae at high exposure levels despite the wide range of possible larval LD_50_ values. We calculated fit to the empirical data based on the number of adults and eggs, but not larvae and pupae, however, and including these latter two endpoints in future analyses may allow better inference on larval toxicity.

We also leveraged our model to predict a more precise threshold dose where statistically significant negative effects on colonies would have occurred, by fitting toxicity parameters from the concentrations tested in the feeding study, then predicting untested concentrations between 50 and 95 µg/kg. This method is more rigorous than simply interpolating responses between tested concentrations because it can account for uncertainty and non‐linear effects or tipping points, and it allows for consideration of statistical significance. The threshold for determining a significant adverse effect, based on prediction intervals, can be adjusted based on the desired level of confidence and a consideration of biological risk. If the 95% prediction interval for reduction in bee count from the control does not contain zero, at least 95% of colonies are predicted to be adversely affected, taking into account between‐colony variance and uncertainty in colony health and substance toxicity. Using the more conservative 68% prediction interval, we estimated a significant effect threshold of 55 µg/kg, based on adverse effects across all endpoints. The 95% and 99% prediction intervals resulted in a significant effect threshold of 80 µg/kg. By comparison, a statistical analysis of the feeding study data found a NOAEC and LOAEC of 19 and 36 µg/kg, respectively, for adults and pupae, and 36 and 72 µg/kg for eggs and larvae (Louque 2016). It is important to consider that our model‐derived significance effect threshold describes the lowest concentration at which ingestion‐induced mortality is expected to begin significantly impacting colony‐level endpoints but does not include other types of effects such as non‐lethal effects or contact exposure that may be better represented by the empirically derived endpoints. Despite this caveat, our analysis shows how colony dynamics models can estimate outcomes at exposure levels that could not be tested due to logistical or financial constraints.

## Conclusion

We challenged the VarroaPop + Pesticide bee colony dynamics model to simulate a publicly available registrant‐submitted data set from a colony feeding study in which colonies were exposed to pesticide‐spiked nectar at six concentrations, and population‐level effects were tracked over several months. We successfully fit the model to these data using Approximate Bayesian Computation with Sequential Monte Carlo, and inferred parameter distributions that best describe the dose‐response relationships and other key colony characteristics. While the model fit well at intermediate exposure levels, it underestimated adverse effects at low exposure (36 µg/kg) and overestimated colony recovery at the highest exposure (140 µg/kg), for the adult and pupa endpoints, which suggests that additional mechanisms beside oral toxicity‐induced mortality may have contributed to colony declines. Our results demonstrate that honey bee colony models, combined with Bayesian model inference, can investigate hypotheses about individual‐level responses to pesticides from ecologically relevant colony‐level data. These parameterized models can also predict how colonies will respond to hypothetical scenarios such as untested concentrations, changes in weather or additional stressors. Our findings suggest that applied colony dynamics models are a promising tool for inference in support of higher‐tier pesticide risk assessments.

## Supporting information

Appendix S1

Appendix S2

Appendix S3

## Data Availability

Input data (Minucci 2021*a*) are available on Figshare: https://doi.org/10.6084/m9.figshare.c.5402901.v1. Scripts (Minucci 2021*b*) to run this analysis are available on Zenodo: https://doi.org/10.5281/zenodo.4721797.
