# Supplementary material for: Inferring pesticide toxicity to honey bees from a field‐based feeding study using a colony model and Bayesian inference
Source: Ecol Appl. 2021 Sep 5;31(8):e02442. doi: 10.1002/eap.2442 (PMC8928141; doi:10.1002/eap.2442)
Supplement: Supplementary file 1 — Appendix S1 [file EAP-31-e02442-s003.pdf]

Supporting Information. Minucci, J.M., R. Curry, G. DeGrandi-Hoffman, C. Douglass, K. Garber, and S.T. Purucker. 2021. Inferring pesticide toxicity to honey bees from a field-based feeding study using a colony model and Bayesian inference. Ecological Applications.

Appendix S1

Table S1: Static VarroaPop+Pesticide parameter values used in this study.

Category	Parameter name	Description	Units	Value
Foraging				
	IPollenTrips	Pollen trips per day	times/day	8
	INectarTrips	Nectar trips per day	times/day	17
	IPollenLoad	Pollen collected per trip	mg	15
	INectarLoad	Nectar collected per trip	mg	30
	ForagerMaxProp	Max. proportion of workers that can be active foragers		0.3
Consumption				
	CL4Pollen	Pollen consumption, worker larvae, age 4 days	mg/day	1.8
	CL5Pollen	Pollen consumption, worker larvae, age 5 days	mg/day	3.6
	CA13Pollen	Pollen consumption, worker adults, days 1–3	mg/day	6.7
	CA410Pollen	Pollen consumption, worker adults, days 4–10	mg/day	6.7
	CA1120Pollen	Pollen consumption, worker adults, days 11–20	mg/day	1.7

	CForagerPollen	Pollen consumption, foragers	mg/day	0
	CLDPollen	Pollen consumption, drone larvae	mg/day	3.6
	CADPollen	Pollen consumption, drone adults	mg/day	2.0
	CL4Nectar	Nectar consumption, worker larvae, age 4 days	mg/day	60
	CL5Nectar	Nectar consumption, worker larvae, age 5 days	mg/day	120
	CA13Nectar	Nectar consumption, worker adults, days 1–3	mg/day	60
	CA410Nectar	Nectar consumption, worker adults, days 4–10	mg/day	140
	CA1120Nectar	Nectar consumption, worker adults, days 11–20	mg/day	60
	CForagerNectar	Nectar consumption, foragers	mg/day	292
	CLDNectar	Nectar consumption, drone larvae	mg/day	130
	CADNectar	Nectar consumption, drone adults	mg/day	225
Demographics				
	RQEnableReQueen	Enable requeening?		off
	EToLXition	% of eggs that successfully transition to larvae	%	100
	LToBXition	% of larvae that successfully transition to pupae (brood)	%	100
	BToAXition	% of pupae (brood) that successfully transition to adults	%	60
	AToFXition	% of adults that successfully transition to foragers	%	100

Table S2: Percent of the study period that each treatment was predicted by our model to have a significant reduction in bee counts, compared to the control, for three different levels of confidence. The study period is the first day of treatment until the final colony condition assessment of 2014. An endpoint was considered significantly reduced (compared to the control) when the prediction interval of the change did not contain zero. Clothianidin levels 50–70 µg/kg and 75–95 µg/kg were predicted by the model but were not present in the empirical feeding study.

Percent of Study Period Significantly Lower Than Control						
	**68% PI**		**95% PI**		**99% PI**	
**Clothianidin exposure**	**Adults**	**Mean all endpoints**	**Adults**	**Mean all endpoints**	**Adults**	**Mean all endpoints**
**10 µg/kg**	0.0	0.0	0.0	0.0	0.0	0.0
**19 µg/kg**	0.0	0.0	0.0	0.0	0.0	0.0
**36 µg/kg**	0.0	0.0	0.0	0.0	0.0	0.0
50 µg/kg	0.0	**0.2**	0.0	0.0	0.0	0.0
55 µg/kg	**13.7**	**8.8**	0.0	0.0	0.0	0.0
60 µg/kg	**40.2**	**33.8**	0.0	0.0	0.0	0.0
65 µg/kg	**45.3**	**43.6**	0.0	0.0	0.0	0.0
70 µg/kg	**54.7**	**50.9**	0.0	0.0	0.0	0.0
**72 µg/kg**	**50.4**	**45.5**	0.0	0.0	0.0	0.0
75 µg/kg	**80.3**	**82.5**	0.0	**1.3**	0.0	0.0
80 µg/kg	**79.5**	**84.4**	**39.3**	**38.7**	**9.4**	**6.4**
85 µg/kg	**80.3**	**87.0**	**49.6**	**47.6**	**21.4**	**14.8**
90 µg/kg	**80.3**	**88.7**	**48.7**	**47.6**	**36.8**	**24.0**
95 µg/kg	**81.2**	**89.3**	**53.0**	**50.0**	**41**	**32.9**
**140 µg/kg**	**95.7**	**95.9**	**78.6**	**84.0**	**55.6**	**52.2**
