# Supplementary material for: Inferring pesticide toxicity to honey bees from a field‐based feeding study using a colony model and Bayesian inference
Source: Ecol Appl. 2021 Sep 5;31(8):e02442. doi: 10.1002/eap.2442 (PMC8928141; doi:10.1002/eap.2442)
Supplement: Supplementary file 2 — Appendix S2 [file EAP-31-e02442-s002.pdf]

Supporting Information. Minucci, J.M., R. Curry, G. DeGrandi-Hoffman, C. Douglass, K. Garber, and S.T. Purucker. 2021. Inferring pesticide toxicity to honey bees from a field-based feeding study using a colony model and Bayesian inference. Ecological Applications.

Appendix S2

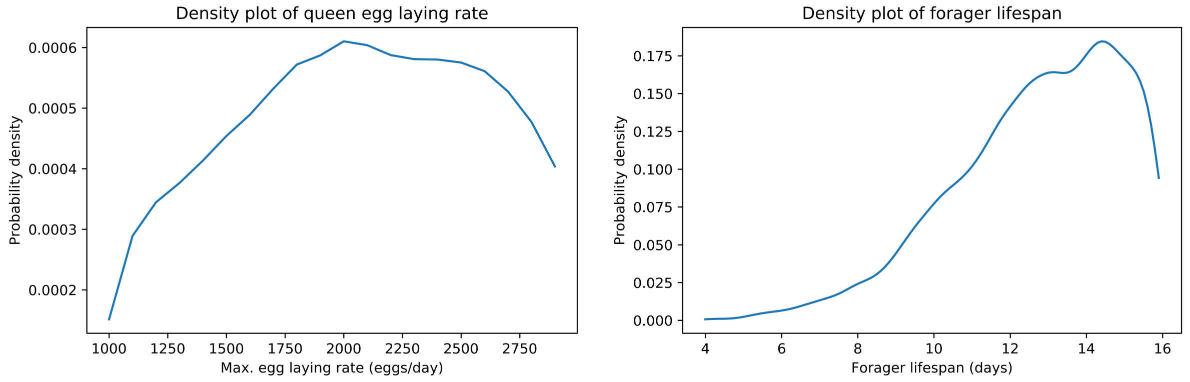

Figure S1: Probability density of maximum queen egg laying rate (eggs/day) and forager lifespan (days) for individual colonies in the feeding study, as predicted from 200 draws of the joint posterior.

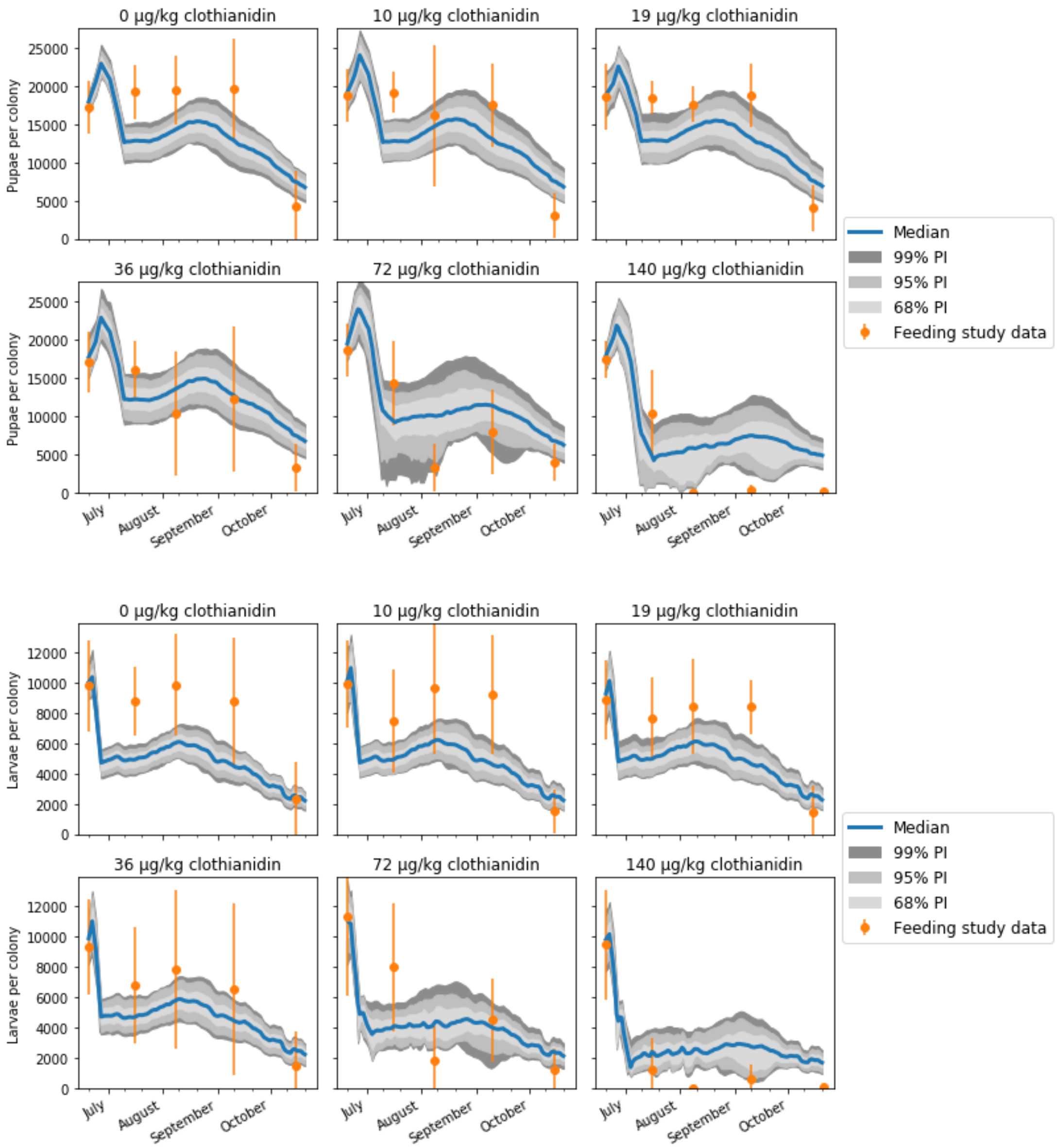

Figure S2: Predicted number of pupae (top) and larvae (bottom) during the feeding study versus the empirical data (orange dots with lines showing standard deviation). Solid blue lines represent the median prediction and shaded regions denote the 68%, 95% and 99% prediction intervals.

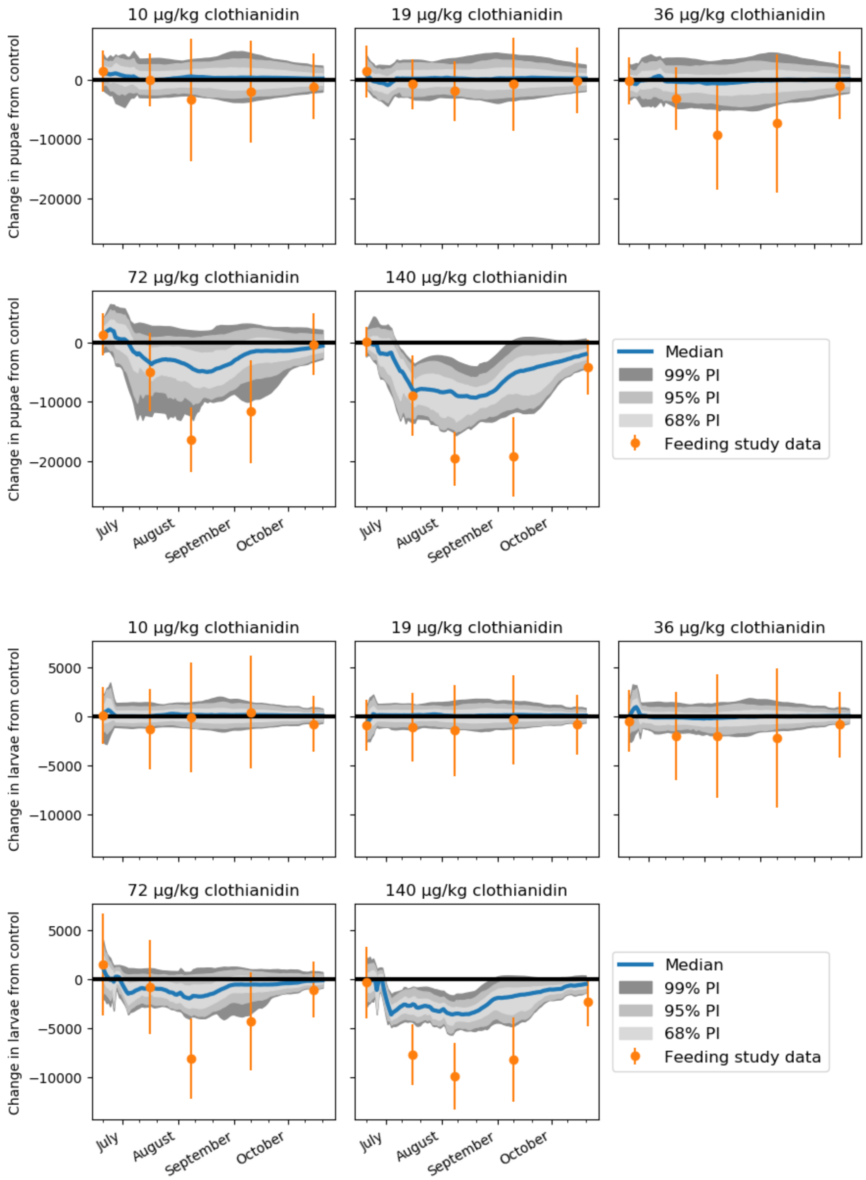

Figure S3: Predicted change in number of pupae (top) and larvae (bottom) from the control. Empirical feeding study data is represented by orange dots. Solid blue lines represent the median prediction and shaded regions denote the 68%, 95% and 99% prediction intervals.

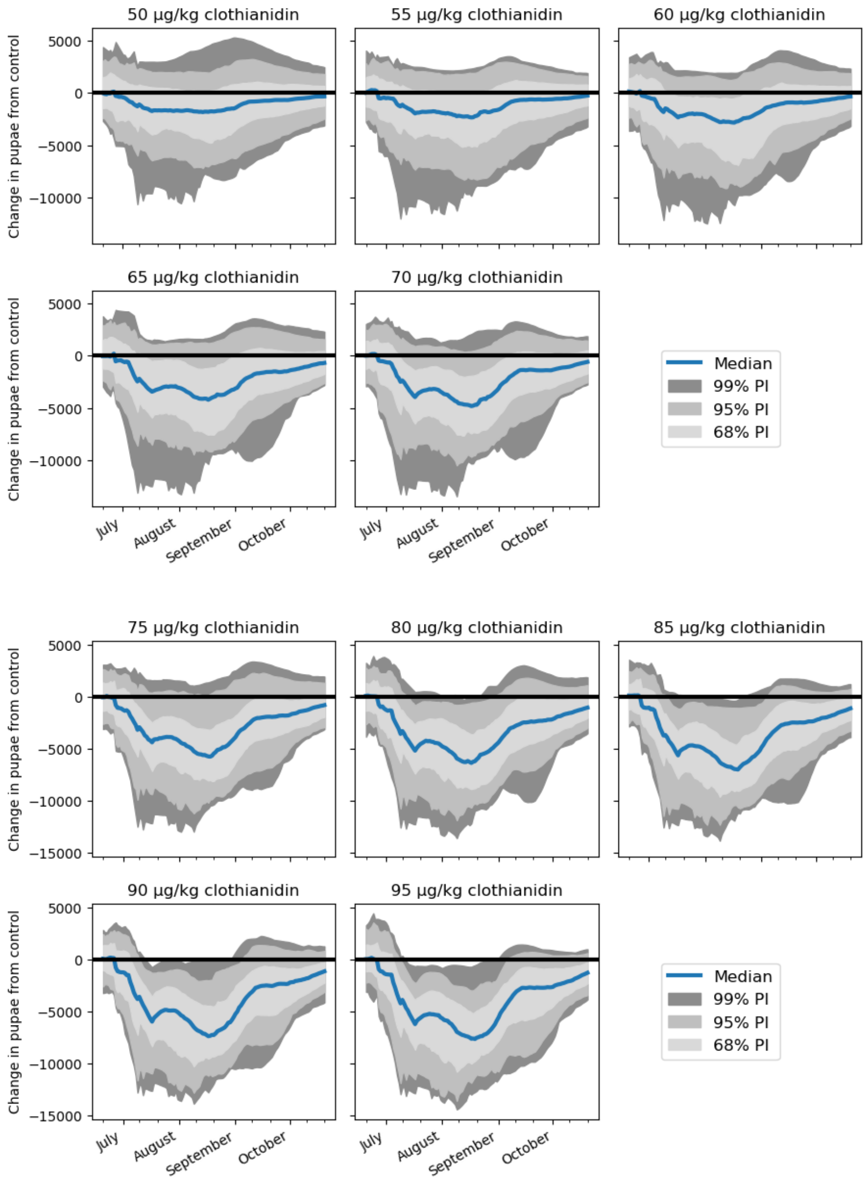

Figure S4: Predicted change, from the control, in number of pupae for clothianidin levels not tested in the feeding study. Solid blue lines represent the median prediction and shaded regions denote the 68%, 95% and 99% prediction intervals.

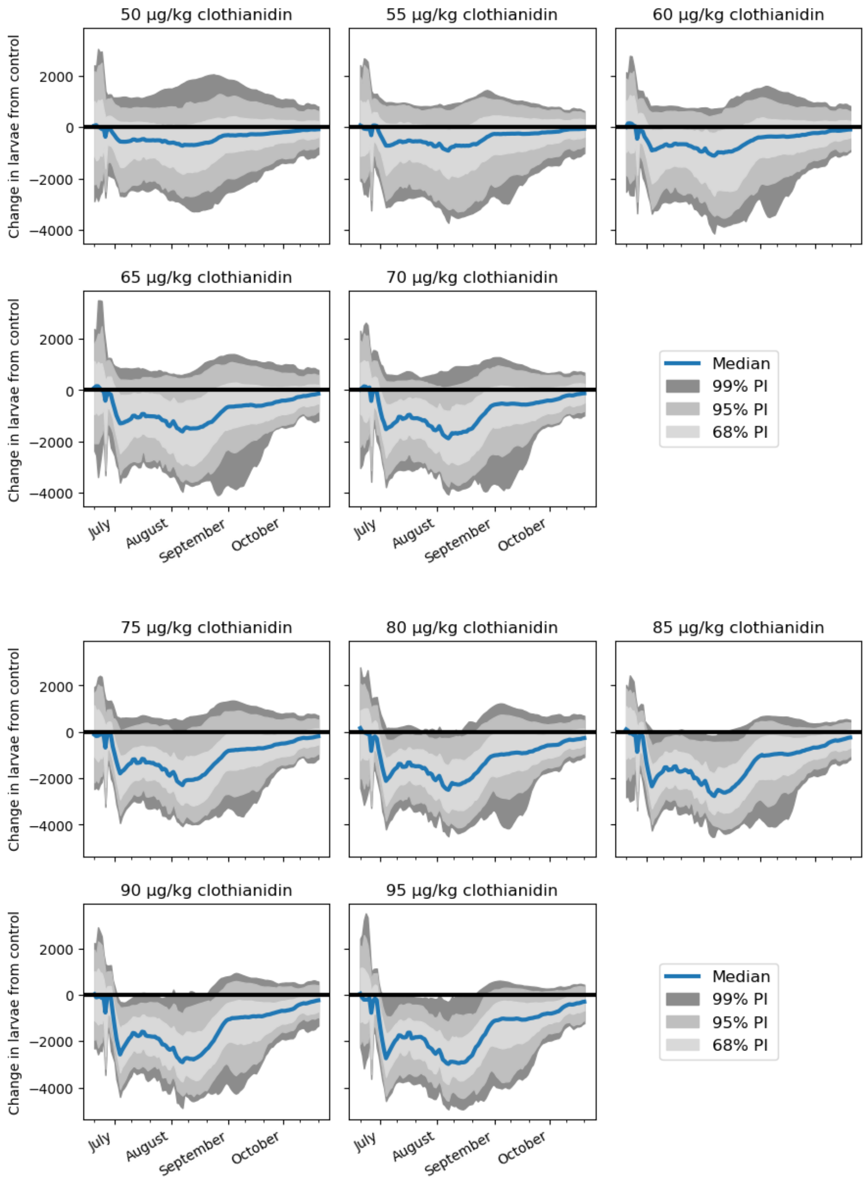

Figure S5: Predicted change, from the control, in number of larvae for clothianidin levels not tested in the feeding study. Solid blue lines represent the median prediction and shaded regions denote the 68%, 95% and 99% prediction intervals.

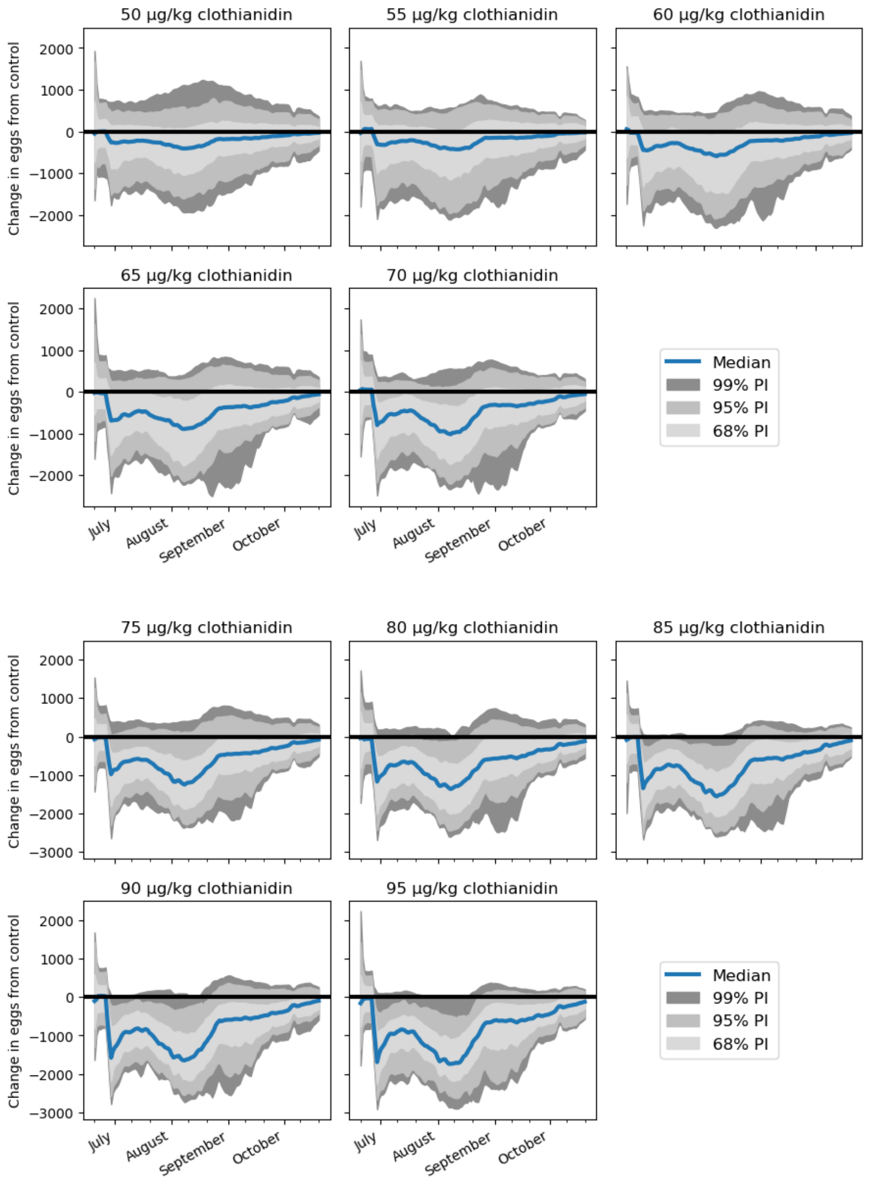

Figure S6: Predicted change, from the control, in number of eggs for clothianidin levels not tested in the feeding study. Solid blue lines represent the median prediction and shaded regions denote the 68%, 95% and 99% prediction intervals.
