# Supplementary material for: Inferring pesticide toxicity to honey bees from a field‐based feeding study using a colony model and Bayesian inference
Source: Ecol Appl. 2021 Sep 5;31(8):e02442. doi: 10.1002/eap.2442 (PMC8928141; doi:10.1002/eap.2442)
Supplement: Supplementary file 3 — Appendix S3 [file EAP-31-e02442-s001.pdf]

Supporting Information. Minucci, J.M., R. Curry, G. DeGrandi-Hoffman, C. Douglass, K. Garber, and S. T. Purucker. 2021. Inferring pesticide toxicity to honey bees from a field-based feeding study using a colony model and Bayesian inference. Ecological Applications.

Appendix S3

Figure S1. Posterior parameter distributions for our VarroaPop+Pesticide-based model, as inferred from the empirical feeding study data. Axes are labeled with parameter names specified in the VarroaPop+Pesticide program (see Table 1 in the main text for definitions). Marginal posterior distributions are shown in the diagonal. All priors were uniform across the x-axis range. Bivariate scatterplots (top half) and heatmaps (bottom half) of the distribution of accepted parameters illustrates covariance between parameters.

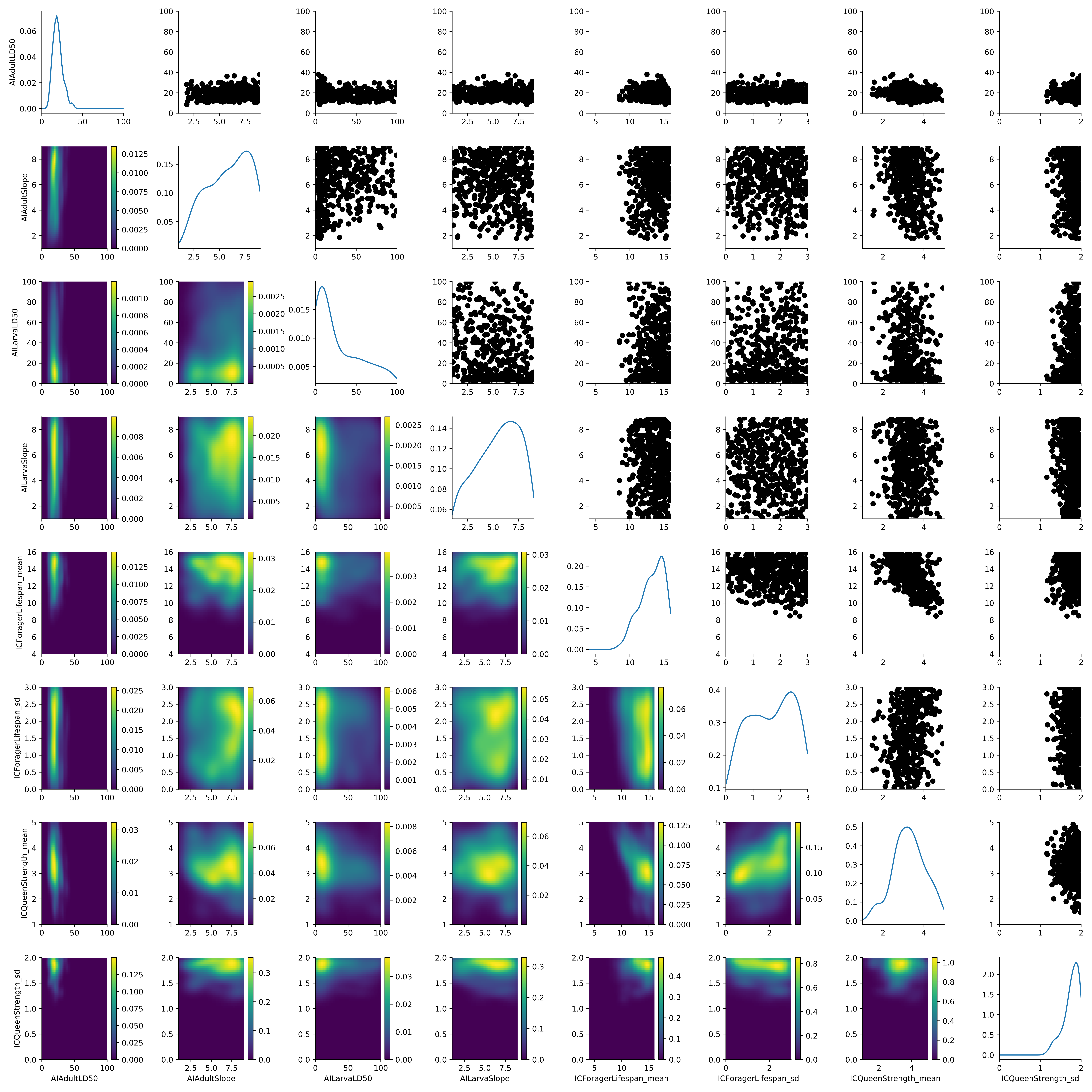
AIAdultLD50
AIAdultSlope
AILarvaLD50
AILarvaSlope
ICForagerLifespan_mean
ICForagerLifespan_sd
ICQueenStrength_mean
ICQueenStrength_sd
AIAdultLD50
AIAdultSlope
AILarvaLD50
AILarvaSlope
ICForagerLifespan_mean
ICForagerLifespan_sd
ICQueenStrength_mean
ICQueenStrength_sd
